# Fiber Optic Sensors Based on the Faraday Effect

**DOI:** 10.3390/s21196564

**Published:** 2021-09-30

**Authors:** Pedja Mihailovic, Slobodan Petricevic

**Affiliations:** School of Electrical Engineering, University of Belgrade, 11000 Belgrade, Serbia; slobodan@etf.bg.ac.rs

**Keywords:** Faraday effect, magnetometry, fiber optic current sensor, temperature compensation

## Abstract

Some 175 years ago Michael Faraday discovered magnetic circular birefringence, now commonly known as the Faraday effect. Sensing the magnetic field through the influence that the field has on light within the fiber optic sensor offers several advantages, one of them fundamental. These advantages find application in the measurement of electric current at high voltages by measuring the induced magnetic field, thus warranting application for this kind of fiber optic sensor (FOS) in future smart grids. Difficulties in designing and manufacturing high-performance FOSs were greatly alleviated by developments in optical telecommunication technology, thus giving new impetus to magnetometry based on the Faraday effect. Some of the major problems in the processing of optical signals and temperature dependence have been resolved, yet much effort is still needed to implement all solutions into a single commercial device. Artificial structures with giant Faraday rotation, reported in the literature in the 21st century, will further improve the performance of FOSs based on the Faraday effect. This paper will consider obstacles and limits imposed by the available technology and review solutions proposed so far for fiber optic sensors based on the Faraday effect.

## 1. Introduction

Humanity’s ever-increasing demand for energy, especially electric energy that has high quality and acceptable distribution losses, is pushing electrical power systems towards higher complexity, voltage levels and transmission capacities. To ensure power quality and decrease losses, smart power grids need a vast number of current sensors, causing increased data flow. Incorporation of renewable energy sources further increases the need for monitoring and control [1]. Fiber optic current sensors (FOCSs), also called optical current transducers (OCTs), have inherent advantages over current transformers, including the following:

- Wider frequency bandwidth;
- Immunity to electromagnetic interferences;
- Absence of saturation effects;
- Possibility of dielectric measuring head with no power supply on high-voltage side;
- Possibility of wavelength division multiplexing (WDM);
- Isolation of sensor electronics from the measuring head by optical fiber (OF);
- Smaller size and weight.

These advantages are becoming more significant.

The Faraday effect (FE) is one of the principles OCT operation can be based on. Fiber Bragg gratings are also proposed [2], utilizing the benefit from high-voltage cables with integrated OF (OPGW/OPPC) that are on the market today [3].

The scope of this paper is limited to FOSs based on the FE. These include applications outside power grids, from the protection of generators to tokamaks [4,5,6], as well as magnetometers. Due to the large scope of design issues researchers have no choice but to place emphasis on one aspect of sensor design in a review paper. For example, the problem of linear birefringence is considered in detail by Wang et al. [7], and a comparison of OF magnetometer performance parameters is presented by Peng et al. [8]. In the approach we have chosen, the FE is thoroughly presented at the second Section which provides a basis for considering problems and solutions of the sensor design. The emphasis of our review is on the temperature compensation methods presented in Section 5. Linearity, measurement range and normalization are discussed in Section 3 and Section 4. Faraday materials (FMs) are discussed in Section 6 and three promising FMs are compared.

The aim of the paper is to inform young researchers about problems and measurement techniques that can solve them when designing FOSs based on the FE. Three main directions of research are presented, but do not cover all possible applications of FOSs based on the FE; we hope, therefore, that this paper will help and motivate researchers to create better FMs and new measurement methods.

## 2. The Faraday Effect

The Faraday effect represents a rotation of the plane of polarization of linearly polarized light while passing through a medium in the presence of a magnetic field. The Faraday angle (FA), θ, is proportional to the component of magnetic flux density parallel to the light beam, $B_{∥}$, the length of the optical path through the Faraday material, l, and the material-dependent Verdet constant, V:

(1) $$\theta =\int_{0}^{l}V\vec{B}\vec{dl}.$$

In a homogenous field and medium the FA is $\theta =VB_{∥}l$. Faraday discovered the effect in 1845 while working with heavy glass [9,10], but later the presence of the effect was confirmed in crystals, liquids [11], gases [12,13] and plasma [14]. Artificial structures possessing Faraday rotation (FR), such as optical fibers [15,16,17,18,19], magneto-optic photonic crystals [20,21], magneto-optic ferrofluids [22] and nano-composite polymers [23] have also been made.

Linearly polarized light is a superposition of equal amounts of right and left circularly polarized modes. Two circularly polarized light waves, ${\vec{E}}_{R}$ and ${\vec{E}}_{L}$, propagating along *z* axes with different propagation constants, $k_{R},\;k_{L}$, are written out as:

(2) $${\vec{E}}_{R}=\frac{E_{0}}{2}\left[{\vec{e}}_{x}\cos\left(k_{R}z-\omega t\right)+{\vec{e}}_{y}\sin\left(k_{R}z-\omega t\right)\right],$$

(3) $${\vec{E}}_{L}=\frac{E_{0}}{2}\left[{\vec{e}}_{x}\cos\left(k_{L}z-\omega t\right)-{\vec{e}}_{y}\sin\left(k_{L}z-\omega t\right)\right].$$

Their superposition is again linearly polarized, (if we assume no absorption):

(4) $$\vec{E}={\vec{E}}_{R}+{\vec{E}}_{L}=E_{0}\left[\cos\left(\frac{\left(k_{R}+k_{L}\right)z}{2}-\omega t\right)\right]\left[{\vec{e}}_{x}\cos\frac{\left(k_{R}-k_{L}\right)z}{2}+{\vec{e}}_{y}\sin\frac{\left(k_{R}-k_{L}\right)z}{2}\right],$$

with the plane of polarization rotated by the half of circular retardation:

(5) $$\theta =\frac{\left(k_{R}-k_{L}\right)z}{2}=\frac{k_{0}\left(n_{R}-n_{L}\right)z}{2}=\frac{\pi \cdot z}{\lambda_{0}}\left(n_{R}-n_{L}\right).$$

Some materials are optically active, and circular birefringence is inherent to them. The Faraday effect is magnetically induced optical activity (OA), or magnetic circular birefringence. Some crystals possess both OA and FR. By definition, the FA is positive for counterclockwise rotation when the magnetic flux density vector has the same direction as the wave vector, and for clockwise rotation when these vectors are of the opposite direction. Therefore, the FE is truly a nonreciprocal effect, and the FA will double after the light is reflected and goes back along the same path. OA, independent of the magnetic field direction, is a reciprocal effect and will cancel out after the light is reflected. The Faraday material (FM) can also be described by the Verdet constant, defined in respect to the magnetic field, $V_{H}=\frac{B}{H}V$. Since data for the relative magnetic permeability of FMs are often unavailable, for their comparison the relation $V_{H}=\mu_{0}V$ is used, where $\mu_{0}$ is vacuum permeability.

Since the real and imaginary parts of the index of refraction must obey Kramers–Kronig relations, the magnetic circular birefringence of the FM means that there is also a magnetic circular dichroism present, and the light at the exit of the FM is actually elliptically polarized with the major axes rotated for the FA. The ratio of the major and minor axis of polarization ellipse is [24,25]:

(6) $$\frac{b}{a}=\frac{e^{-\alpha_{R}}-e^{-\alpha_{L}}}{e^{-\alpha_{R}}+e^{-\alpha_{L}}},$$

and since magnetic circular dichroism is weak (absorption coefficients of circular modes are almost equal, $\alpha_{L}\approx \alpha_{R}$), eccentricity is close to one and polarization is almost linear.

If we assume that, at optical frequencies, the relative magnetic permeability is close to one [26,27], OA and the FE can be phenomenologically described through the tensor of dielectric permittivity, ε**,** or the tensor of conductivity, σ. The derivation of the linear state of the polarization rotation angle for an isotropic material is presented in Appendix A. According to Equation (A26) the total rotation is, approximately, the superposition of OA and FR:

(7) $$\theta_{tot}=\frac{1}{2}\sqrt{\frac{\mu_{0}\omega^{2}}{\varepsilon_{d}}}\left(\varepsilon_{xy}^{\left(0\right)}+\varepsilon_{xy}^{\left(1\right)}B\right)l=\theta_{0}+VBL.$$

Verdet constant is therefore proportional to $\varepsilon_{xy}^{\left(1\right)}$ term:

(8) $$V=\frac{1}{2}\sqrt{\frac{\mu_{0}\omega^{2}}{\varepsilon_{d}}}\varepsilon_{xy}^{\left(1\right)}$$

Propagation through materials possessing both FR and birefringence was analyzed by Ramachandran and Ramaseshan [28] and Tabor and Chen [29], but their results are actually more general and can be applied to materials that have uniform linear and circular birefringence, regardless of the cause [30]. A distributed parameter model and simulation of light polarization states have been done by YanSong et al. [31] If the medium is birefringent, $\varepsilon_{xx}\neq \varepsilon_{yy}$, two orthogonal elliptical modes exist:

(9) $$\left(\begin{array}{c} E_{x}^{1}\\ E_{y}^{1} \end{array}\right)=E_{01}\left(\begin{array}{c} 1\\ j\Pi \end{array}\right)exp\left[j\left(\omega t-k_{+}z\right)\right],$$

(10) $$\left(\begin{array}{c} E_{x}^{2}\\ E_{y}^{2} \end{array}\right)=E_{02}\left(\begin{array}{c} 1\\ \frac{-j}{\Pi } \end{array}\right)exp\left[j\left(\omega t-k_{-}z\right)\right],$$

where

(11) $$\Pi =\frac{2\varepsilon_{xy}}{\varepsilon_{xx}-\varepsilon_{yy}-\sqrt{{\left(\varepsilon_{xx}-\varepsilon_{yy}\right)}^{2}+4\varepsilon_{xy}{}^{2}}}.\;$$

The resulting light wave is elliptically polarized. A medium possessing birefringence cannot rotate the plane of polarization 90°, and the FR cannot be described by the Verdet constant. As Forman and Jahoda showed [32], the modulation depth for FR measurement is decreased, and new nonlinearity is introduced. For weak optical rotation and birefringence, the phase difference can be approximated as [29]:

(12) $$\Delta k=\sqrt{4\rho^{2}+\eta^{2}},\;$$

where ρ is the rotation per unit length in the absence of birefringence and η is birefringence per unit length in the absence of rotation. Birefringence also complicates temperature dependence [33]. The general conclusion is that birefringent materials should be avoided if possible for sensing purposes, or that birefringence should be compensated for [34,35,36,37,38,39,40]. Unfortunately, birefringence is inevitable in the coiled optical fiber (OF), and stress or the Pockels effect can induce birefringence in crystals. The Pockels effect will induce birefringence in crystals that do not possess central symmetry [41], and this will create measurement error in the presence of an electric field. On the other hand, a polarization state is determined by both circular and linear birefringence, and there are propositions for the measurement of both simultaneously [42,43,44]. After the linear birefringence is calculated, it can be used for temperature compensation if temperature shift is the cause, or for electric field calculation if an electric field is the cause, but not both. In some crystals, such as Bi_12_GeO_20_, optical rotatory power can be very strong [45,46], and the approximation given by Equation (12) is not valid. OA can be canceled out in the reflexive configuration or it can be used for temperature compensation [24].

Calculation of the Verdet constant comes down to calculation of the term $\varepsilon_{xy}^{\left(1\right)}$ or, equivalently, $\sigma_{xy}^{\left(1\right)}$. For example, in the single-particle model of plasma, $\varepsilon_{xy}^{\left(1\right)}B=-\varepsilon_{0}\frac{\omega_{p}^{2}\omega_{B}}{\omega \left(\omega^{2}-\omega_{B}^{2}\right)}$, where $\omega_{p}=\sqrt{\frac{n_{e}e^{2}}{m_{e}\varepsilon_{0}}}$ is plasma frequency and $\omega_{B}=\frac{e}{m_{e}}B$ is cyclotron frequency, the FA is $\theta =\frac{1}{2}\sqrt{\frac{\mu_{0}\omega^{2}}{\varepsilon_{0}}}\varepsilon_{xy}^{\left(1\right)}Bl=-\frac{1}{2c}\frac{\omega_{p}^{2}\omega_{B}}{\omega^{2}-\omega_{B}^{2}}l=-\frac{1}{2c}\frac{n_{e}e^{3}}{m_{e}^{2}\varepsilon_{0}}\frac{1}{\omega^{2}-\omega_{B}^{2}}Bl$, and for small fields when light frequency is much higher than cyclotron frequency $\left(\omega \gg \omega_{B}\right)$, the FA follows the lambda-squared law often used in astronomy [47,48]:

(13) $$\theta \approx \frac{e^{3}}{8\pi^{2}m_{e}^{2}\varepsilon_{0}c^{3}}\lambda_{0}^{2}n_{e}Bl.$$

Modeling of the Verdet constant in the solid state was first conducted by Becquerel [49], using the classical theory of the Zeeman effect. He showed the rotation to be linearly dependent on the optical dispersion:

(14) $$V=\frac{\left|e\right|\lambda_{0}}{2m_{e}c^{2}}\cdot \frac{\partial n}{\partial \lambda }.\;$$

Born and Jordan [50], using the quantum approach to the dispersion relation in the presence of a magnetic field, showed that the Becquerel relation is valid for the diamagnetic part of the Verdet constant. The diamagnetic FE exists in all solids and originates from Zeeman splitting. They also comment that there is no paramagnetic contribution to Faradays rotation in diamagnetic materials. The diamagnetic part is temperature independent for moderate fields but not to low temperatures ($\mu_{B}B\ll k_{B}T$, where $\mu_{B}$ is the Bohr magneton), and the paramagnetic part is approximately inversely proportional with temperature. In the quantum treatment of the problem, the result depends critically on the nature of the medium. All of the electrons in a solid contribute to FR, but on optical frequencies the influence of the conduction electrons is dominant [51]. A magnetic field can induce FR mainly through two mechanisms [27,52]: Zeeman splitting of the energy levels—diamagnetic FR, and changing the density matrix elements—paramagnetic FR. For solids with cubic symmetry, Bennet and Stern showed [51] that the diamagnetic part is proportional to $\frac{\omega_{\beta \alpha }}{{\left(\omega_{\beta \alpha }^{2}-\omega^{2}\right)}^{2}}$ and the paramagnetic part to $\frac{1}{\omega_{\beta \alpha }^{2}-\omega^{2}}$. Despite there being several other approaches of modeling for different materials [27,51,52,53,54,55,56,57], the main conclusions that were important from a sensing point of view, and experimentally verified, can be deduced from Bennet and Stern’s paper:

1. The Verdet constant is highest in the vicinity of the absorption line ($\omega \to \omega_{\beta \alpha }$). Therefore, magneto-optical quality is introduced as a ratio of the Verdet constant and absorption, $\chi =\frac{V}{\alpha }$ [58,59]. This parameter expresses material usability as a sensor for the Faraday effect. Since it is wavelength-dependent, for sensing purposes a light source should be chosen with a wavelength where the magneto-optical quality has its maximal value [60,61,62];
2. Far from the absorption line, the paramagnetic FE will dominate and can be, for example, 20 times stronger than diamagnetic FE, as shown for rare-earth oxide glasses [61], or can even be three orders of magnitude stronger [27];
3. Since two parts have different temperature dependences and different spectral dependences, temperature dependence is wavelength-dependent. The Verdet constant decreases with temperature and for most of the FMs can be modeled as $V={\mathbb{C}}_{1}+{\mathbb{C}}_{2}T^{-{\mathbb{C}}_{3}},\;{\mathbb{C}}_{3}\in \left(0,1\right)$, where ${\mathbb{C}}_{1},\;{\mathbb{C}}_{2},\;{\mathbb{C}}_{3}$ are wavelength-dependent [24,63,64];
4. Diamagnetic FR is symmetrical around a resonant frequency and the paramagnetic FR is antisymmetric.

Paramagnetic FR can experience saturation for strong fields [65], but in a magnetometry field is usually far below this limit. The inverse FE represents magnetization of the material when exposed to intense, circularly polarized light [66].

## 3. Faraday Effect Magnetometry and Electrical Current Sensing

The FE provides the possibility to measure the magnetic field or electrical current that induces the field. FOSs can be divided into intrinsic and extrinsic types. In the intrinsic type, light stays inside the OF, which is a sensor and communication channel. In the extrinsic type, light exits the OF to be modulated outside of it and again coupled to another OF that carries light to the detector. A magnetic field sensor has to be an extrinsic FOS, since it is sensitive to $\int^{\;}\vec{B}\vec{dl}$ and the OF would have to trace magnetic field lines, unknown at the beginning of measurement. OCTs can be constructed as extrinsic or intrinsic FOSs.

The fundamental advantage of FE magnetometry is that only FMs and photons are indispensable inside the field. Since FMs can be dielectric, this is the only kind of magnetometry without metals or semiconductors in the field, and the perturbation of the measured field is minimal. Submillimeter spatial resolution is possible with new FMs.

**Figure 1:**
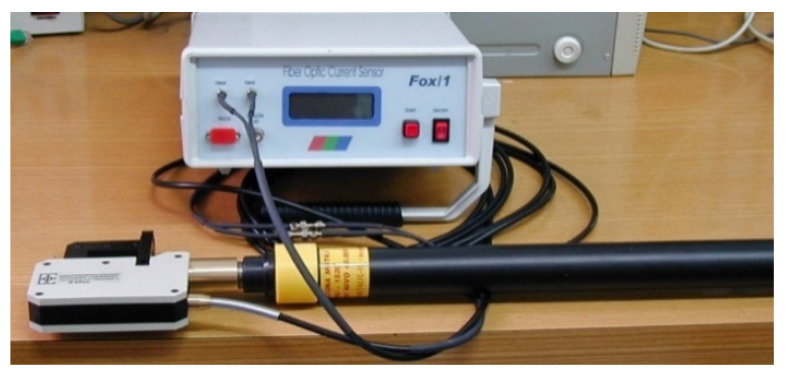
Portable OCT with measurement head mounted on an insulating rod certified to operate up to 100 kV.

Advantages of OCTs in the monitoring of power systems are also significant [5,67]. Since the FE response time is in the range of ns or less, the frequency range is practically limited by the optoelectronic conversion block. Owing to its wide frequency bandwidth, an OCT is able to detect transient electrical faults in power systems [68,69,70]. Light is the carrier of information so, in contrast to metallic wires, electromagnetic induction is not a problem, which is also important in power systems [71]. Sensors can be designed small, portable, safe and easy to operate and maintain. With an extrinsic OCT output, an OF carries the information on the current in the form of intensity-modulated light, and WDM can be used to carry this information through the same OF used for other FOSs in the system (for example, FBG used for temperature monitoring). Unlike current transformers, extrinsic OCTs can be applied without interruption of the power supply. High electric insulation is mentioned in almost every paper introduction, and instead of referencing these we will display, in Figure 1, the measurement head, mounted on an insulating rod certified to operate up to a 100 kV voltage level.

There are several obstacles as well. From the engineering point of view normalization, nonlinear transfer function, limited measurement range and cross sensitivity to temperature, electrical field and vibrations are the main problems to be solved.. Solutions are presented later in the text. The economic obstacle is yet to be resolved.

**Figure 2:**
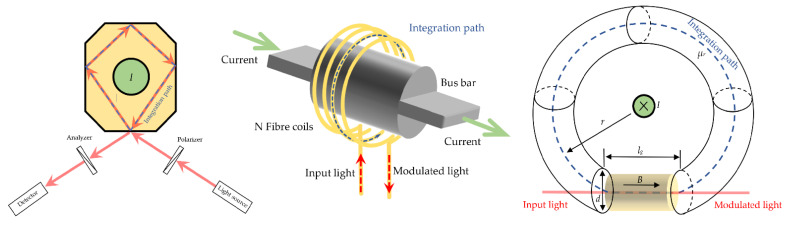
Methods for sensing electrical currents from left to right: a closed optical path through a Faraday material, a closed optical path through OF possessing FR and a magnetic concentrator encircling conductor with FC inside the gap.

Current sensing differs from magnetometry because it is necessary to suppress all the magnetic field sources but one, a conductor, the current of which we wish to measure. This can be achieved in two ways: by a closed optical loop around the conductor [72,73,74], (a) and (b) in Figure 2, or by a magnetic ring concentrator encircling the conductor [35,75,76,77], (c) in Figure 2. FR and current are connected through Ampere’s law. In Figure 2, the integration path, L, is depicted in blue color and the optical path in red.

For homogeneous crystals the FA is (a) $\theta =\oint^{\;}V\vec{B}\vec{dl}=\mu_{0}V\oint^{\;}\vec{H}\vec{dl}=V_{H}I$, and for *N* curls around the conductor the FA is (b) $\theta =\oint^{\;}V\vec{B}\vec{dl}=\mu_{0}V\oint^{\;}\vec{H}\vec{dl}=NV_{H}I$. For the concentrator, (c), $I=\oint \vec{H}\vec{dl}=\int_{l}\vec{H}\vec{dl}+\oint_{L-l}\vec{H}\vec{dl}=\frac{1}{\mu_{0}\mu_{r}^{c}}\int_{l}\vec{B}\vec{dl}+\frac{1}{\mu_{0}\mu_{r}}\int_{L-l}\vec{B}\vec{dl}=\frac{\theta }{\mu_{0}\mu_{r}^{c}V}+\frac{1}{\mu_{0}\mu_{r}}\int_{L-l}\vec{B}\vec{dl}\Rightarrow \theta =\mu_{r}^{c}V\left(\mu_{0}I-\frac{\mu_{r}^{c}}{\mu_{r}}\int_{L-l}\vec{B}\vec{dl}\right)$. If the relative permeability of an FM is much smaller than the relative permeability of a concentrator ($\mu_{r}^{c}\ll \mu_{r}$), the FA reduces to $\theta \approx \mu^{c}VI=V_{H}I$.

Bulk crystal solutions with multiple closed optical paths around the conductor have been proposed [78,79]. Sensitivity is increased by the increased number of the closed optical paths, N. FM inhomogeneity and reflection-induced retardances break the symmetry of Ampere’s law and cancel perfect EMI immunity [80,81]. FMs with openings have been proposed with the intention to design portable measuring heads [82,83]. A large, homogeneous FM is necessary for this solution, making it expensive. More effective is the intrinsic solution where an OF exhibiting the FE is coiled around a current conductor [7,84,85]. The number of windings, N, determines sensitivity. One drawback of this method is an inevitable birefringence in the bent OF [86,87], which is temperature dependent [88]. The measurement head of the intrinsic type cannot open to envelop the conductor, preventing the design of a portable sensor. Low-birefringence OFs as twisted [40,89,90], annealed [91,92,93] or both [94] have been developed for OCTs. Birefringence disturbances can also be suppressed by more complex setup and signal processing. For example, Ren and Robert suggested alternating coupling of linearly and circularly polarized light to obtain two results, and to calculate FR and birefringence this way [95].

With an openable magnetic ring concentrator, a current clamp for high voltage levels can be designed due to optical isolation. Drawbacks to this method are nonlinearities in the transfer function, introduced by ferromagnetic material and a sizeable measurement head. Soft ferromagnetic materials are recommended to decrease hysteresis nonlinearities. With this extrinsic solution, an OF transmits information in the form of intensity-modulated light; therefore, no special OFs are necessary. A sensing crystal is embedded into the magnetic ring gap, while OFs go through the ferromagnet. Beside suppression of the external magnetic field sources, the magnetic ring serves as a concentrator of magnetic field lines, and increases modulation depth with a factor greater than $3\mu_{r}^{c}$, where $\mu_{r}^{c}$ is the relative permeability of the FM [96]. Special care has to be taken with the design of openable concentrators, since the point of opening can cause vibrations in AC current measurements, creating additional frequency-dependent air gaps. This will spoil the frequency response of the sensor in the vicinity of concentrator mechanical resonance if vibrations are not removed. This can be done by the mechanical construction of concentrator openable sideways. Increase in the magnetic concentrator cross-section area increases the modulation depth, but note that the concentrator decreases the effective safety distance between three-phase conductors in the transformer stations. Although the concentrator is very effective in suppressing outer sources of field, there is a slight dependence (up to 4%) of modulation depth on the conductor position inside the magnetic concentrator [75,76]. A plastic conductor holder inside the concentrator can ensure that conductor position during measurement is the same as the position during calibration [76]. Holder can also secure that conductor is perpendicular to the plane of concentrator keeping the $B_{∥}l$ product maximal. The longer crystal increases the FA, but more light is absorbed and a longer gap in the magnetic circuit is required. It is possible to optimize FC length for maximal modulation depth in the function of magneto-optical quality and the concentrator cross-section area. Instead of using longer crystals, the optical path can be lengthened by multiple reflections without increasing the gap [35,75,97]. If a portable sensor for a power system is designed, a solution with a magnetic ring concentrator imposes itself due to the simplicity and low price.

Power is the only property of light that can be directly measured; therefore, modulation of light polarization has to be converted into light intensity modulation, and that can be done in a polarimetric or interferometric way.

The polarimetric setup uses an analyzer with transmission axes at the angle φ (CCW) in respect to transmission axes of the polarizer for this conversion. Using Malus’ law, we obtain irradiance after the analyzer:

(15) $$Г\left(B\right)={Г}_{0}\cos^{2}\left(\varphi -VBl\right)$$

where ${Г}_{0}$ is irradiance in front of the FM. Voltage after a photodiode is connected into the transimpedance stage is:

(16) $$U\left(B\right)=\beta P_{0}\cos^{2}\left(\varphi -VBl\right)=\frac{\beta P_{0}}{2}\left(1+\cos\left(2\varphi -2VBl\right)\right),$$

where β is a constant that includes all optical losses, as well as the optoelectronic conversion efficiency, and $P_{0}$ is the power of the light source. The optimal angle, φ, for a small signal, which places an optical quiescent point for maximal sensitivity can be found as:

(17) $$\frac{\partial }{\partial \varphi }\left(\frac{\partial U\left(B\right)}{\partial B}\right)=2\beta P_{0}Vl\cos\left(2\varphi -2VBl\right)=0,\;VBl\to 0,\;\varphi =\frac{\pi }{4}.\;$$

The transfer function is then:

(18) $$U\left(B\right)=\frac{\beta P_{0}}{2}\left(1+\sin\left(2VBl\right)\right)=U_{0}+\Delta U\left(B\right).\;$$

If an FM possesses OA, keeping in mind the superposition of OA and FR, the condition for the optimal angle changes to $\varphi =\frac{\pi }{4}+\theta_{0}$.

Interferometric configurations measure the phase difference of two circularly polarized modes by changing them into linear polarizations and letting them interfere at the polarizer. Interrogation can be done with any type of interferometer, but a Sagnac interferometer is the natural idea, where the FE phase shift replaces the Sagnac phase shift, which is also truly nonreciprocal. An analogy with a fiber optic gyroscope (FOG) is full for setup with counter-propagating waves [90,98,99], and solutions developed for a FOG can be applied, providing sensing of the μrad phase difference [58,100]. Phase shifts of non-reciprocal effects, such as Sagnac, are indistinguishable from the FE phase shift [101] but the rotation of OF coils is highly unlikely. Shupe effect errors are common to FOGs and OCTs. A co-propagating setup is favored because of lower sensitivity to asymmetric, time-varying disturbances from the mechanical and thermal domains [102]. Frosio and Dandliker demonstrated an intrinsic reciprocal reflection interferometer, which suppresses reciprocal disturbing effects [103] and doubles the optical path and FA. Co-propagating circular modes are reflected at the OF end and their states of polarization are swapped. Maximum sensitivity can be achieved by imputing a quarter-wave plate, which is a homodyne technique [104]. The temperature dependence of quarter-wave plates has to be solved, and polarization cross-coupling as a consequence of nonideal optical components appears [105]. The polarization cross-coupling can be reduced by the usage of a low-coherence source. Sagnac interferometer configuration with a 3 × 3 directional coupler was also proposed [106,107], but equal amounts of orthogonal circular states have to be coupled to sensing arms, which diminishes the desired simplicity. Recently, polymeric integrated waveguide components were used to perform homodyne detection at 1550 nm [108,109]. Heterodyne detection can also be incorporated in the same manner as with a FOG by introducing a phase modulator and a phase-locked loop amplifier. Heterodyne detection solves the problem of normalization but limits the frequency range. Derivation of the sensor transfer function for this case is presented in Appendix B. If the feedback electronics that control the phase modulator keep the sensor in the point of maximum sensitivity, the response is linear and the measurement range is limited by modulator properties rather than transfer function. A high-frequency carrier signal can be generated in several ways [100,110,111,112,113,114,115]. Temkina et al. [116,117] recognized the problem of economic competitiveness and proposed a solution for the temperature dependence of quarter wave plates based on signal processing. Additionally, the piezoelectric phase modulator was replaced by an electro-optical modulator, shifting the carrier frequency to gigahertz range and decreasing the required length of expensive polarization-maintaining OF. Garcia et al. demonstrated a cost-effective solution that also included a novel FM [118]. With sensitivity determined by the number of OF coils and temperature dependence solved, vibrations are the only problem for a reciprocal interferometer with heterodyne detection, and this is probably the best solution for static OCTs in power systems.

Alternative methods for state of polarization detection have been developed that use a radial grating polarizer [119], Newton’s ring grating [120] or a wedge crystal [121] to convert the state of polarization into a spatially dependent irradiance recorded by a digital camera. The state of polarization can be obtained by image processing. A rotating analyzer was also proposed [122] for educational purposes.

In the majority of experiments with the FE, monochromatic light sources are used, but polychromatic lights have also been proposed [97,123].

## 4. Normalization

The FA is typically small, below 1° for most FMs in the mT range of fields. Light source intensity fluctuations as well as variable absorption in the medium can mask the useful signal entirely. Normalization is the elimination of the influence of light source variation. It can be done by measuring the light source power locally and dividing the sensor output with the result. Another proposed method, called AC/DC, is typically used for slowly varying fields. The ratio $\frac{\Delta U\left(B\right)}{U_{0}}=\sin\left(2VBL\right)$ does not depend on light source intensity. In order to separate ΔU(B) from $U_{0}$, however, which is field independent but time varying, one has to know the frequency range of the measured field. Furthermore, frequency components of $U_{0}$ that overlap with the measured field spectrum cannot be filtered out.

**Figure 3:**
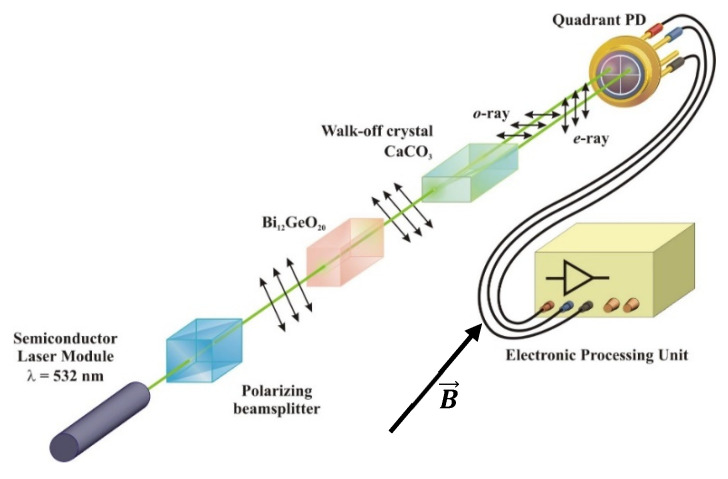
Free-space setup for Faraday angle measurement by the ΔΣ normalization method.

Superior to the mentioned methods is ΔΣ normalization, which does not limit the frequency range and additionally compensates variable losses on the optical path up to the point of splitting of linear polarization modes [124]. The easiest way to explain ΔΣ normalization is by analyzing the free-space setup shown in Figure 3.

The plane of polarization of light after FMs in the absence of the field is set to ±45° in respect to the fast and slow axes of birefringent crystal. Orthogonal polarizations are spatially separated by birefringent crystals, and both depend on light source power in the same way. After transimpedance stages the voltages of the two channels are:

(19) $$U_{1}=\frac{\beta_{1}P_{0}}{2}\left(1+\sin\left(2VBL\right)\right)$$

(20) $$U_{2}=\frac{\beta_{2}P_{0}}{2}\left(1-\sin\left(2VBL\right)\right).\;$$

Using a quadrant photodiode, $\beta_{1}$ and $\beta_{2}$ can be almost perfectly matched, $\beta_{1}=\beta_{2}$, and the calculated FA and magnetic induction are independent of $P_{0}$:

(21) θ=12sin−1(U1−U2U1+U2)=12sin−1(ΔΣ), B=12Vlsin−1(ΔΣ).

The transfer function is nonlinear and sensitivity decreases with an increase in the magnetic field. The measurement range is limited by the lowest acceptable sensitivity rather than by the $B=\frac{\pi }{4Vl}$ condition. A narrow measurement range is an inherent feature of an FE-based FOS due to the nature of the transfer function. A negative feedback technique, used for other types of magnetometers [125], can solve problems of dynamic range and linearity. Applying a feedback magnetic field that exactly opposes the measured field keeps the optical quiescent point fixed. One hesitates to use this technique since it cancels out other FOS advantages. Another possibility is interferometric heterodyne detection, which can also be implemented with bulk FMs [84,111] with increased complexity and cost. Willsch demonstrated an extension in the measuring range using two wavelengths [126].

Polarization fluctuations can be converted into intensity fluctuations by the polarizer placed just in front of the FM. So, the ΔΣ method also suppresses polarization fluctuations at the input optical path, regardless of their origin.

Note that there are two more potential problems: different losses after splitting cannot be compensated, and the background light can spoil normalization since it cancels out in the numerator but not in the denominator of Equation (21). Background light will not exist in the FOS but stray light reflected at the sides of the crystal has the same effect. It loses information carried by its polarization but still contributes to the denominator in ΔΣ normalization. Without additional optics for fiber coupling, maximal crystal length, $l_{MAX}$, is limited by the condition that sideways reflected light cannot couple with output fiber:

(22) $$l_{MAX}=\left(D-2r\right)\sqrt{{\left(\frac{n_{FM}}{NA}\right)}^{2}-1},\;$$

where D is the smallest transversal dimension of FC, r is the OF core radius, $n_{FM}$ is the FM index of refraction and NA is numerical aperture of OF. If duplex OF in one jacket is used for FOS output channels, losses on output optical paths are also matched as much as possible. Polarizing beam splitters are large and impractical for incorporation in FOSs but can be avoided with a slight deviation from the exact solution by placing two analyzers with orthogonal transmission axes behind the FM [127]. Bohnert et al. used an integrated optic polarization splitter to implement ΔΣ normalization with intrinsic OCTs [128]. It is also possible to use polarization-maintaining OF and to convert to intensity modulation in front of the detector [33], but this is not recommendable since the state of polarization is more sensitive to external influence than irradiance.

Mechanical stress and vibrations will also influence light power at the end of output OF through the connectors and macrobending of OF. Niewczas and McDonald proposed two counter-propagating beams through the FM and subtraction of results for two FAs [129]. Modulations caused by the FE are of the opposite sign due to its nonreciprocity, but modulations caused by vibrations are of the same sign and will cancel out by subtraction if counter-propagating beams are equal in power.

An interferometric solution with heterodyne detection uses the fact that the amplitudes of all harmonics are proportional to the power of light incident on the detector and the ratio of harmonics amplitudes is independent of light source intensity and all losses. One drawback is the limitation of frequency bandwidth to range is inferior to the modulation frequency of the carrier.

## 5. Temperature Compensation

Temperature can influence sensor response through:

1. Change of the Verdet constant of an FM with temperature, $\frac{\partial V}{\partial T}$;
2. Change of optical path length through an FM, $\frac{\partial l}{\partial T}$;
3. Change of wavelength of optical source with temperature, $\frac{\partial V}{\partial \lambda }\frac{d\lambda }{dT}$;
4. Change of optical quiescent point with temperature, $\frac{\partial \varphi }{\partial T}$, if an FM possess OA;
5. Change of properties of optical components with temperature (for example, quarter-wave plate);
6. Temperature gradients in OFs.

A ferromagnetic concentrator did not affect temperature dependence in our experiments.

Items 5 and 6 are significant for an intrinsic interferometric solution, where more care should be paid to temperature dependences of other optical components than to temperature dependence of sensing OFs.

If the FM used possesses OA (item 4) its temperature change will influence a response through the shift of the optical quiescent point, since $\frac{\partial \varphi }{\partial T}=\frac{\partial \theta_{0}}{\partial T}$ [71]. FR is much smaller than optical rotatory power even for strong fields. For example, a B_12_GeO_20_ crystal with optical rotatory power $\rho \approx 100\pi \frac{rad}{m}$ and a Verdet constant $V\approx 70\frac{rad}{Tm}$ [45,46] has the ratio of FR to OA $\frac{\theta }{\theta_{0}}=0.22\frac{1}{T}B$. Even moderate OA temperature dependence will have a decisive influence on overall temperature dependence. Therefore, OA has to be removed by design [130] or incorporated into temperature compensation, as explained later.

If the FM used does not possess OA, the relative change of sensor response with temperature for the ΔΣ method is:

(23) 1ΔΣ∂(ΔΣ)∂T=2θcos(2θ)sin(2θ)1θ∂θ∂T,

and for small FA comes down to:

(24) 1ΔΣ∂(ΔΣ)∂T≈1θ∂θ∂T=1V(∂V∂λdλdT+∂V∂T)+1l∂l∂T, 

If wavelength for maximal magneto-optical quality is chosen it is close to the absorption line and the Verdet constant is strongly wavelength dependent, thus making the $\frac{d\lambda }{dT}$ term a problem (item 3). In order to minimize the effect of the $\frac{\partial V}{\partial \lambda }$ term, a temperature-stabilized light source is mandatory, and in that case temperature dependence reduces to the Verdet constant temperature dependence. Alternatively, source wavelength changes can be compensated [131], allowing the usage of low-cost light sources without temperature control.

For FMs with a high Verdet constant, the temperature-induced relative change of FM length (item 2), $\frac{1}{l}\frac{\partial l}{\partial T}$, is two orders of magnitude lower than the temperature-induced relative change of the Verdet constant, $\frac{1}{V}\frac{\partial V}{\partial T}$, and can be neglected in Equation (24). For example, the Bi_12_GeO_20_ crystal thermal expansion coefficient is $16.8\times 10^{-6}K^{-1}$ [132] and the relative thermal change of the Verdet constant at 273 K is $3.8\times 10^{-4}K^{-1}$ [133], making the $\frac{1}{l}\frac{\partial l}{\partial T}$ insignificant.

**Figure 4:**
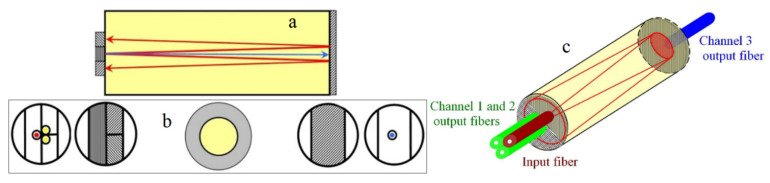
Measuring head that provides 2 channels (green) for the implementation of ΔΣ normalization, and the third channel (blue) for temperature compensation by OA measurement. (**a**) Longitudinal cross-section, (**b**) transversal cross-sections and (**c**) schematic diagram.

The diamagnetic part of the Verdet constant is approximately temperature independent, but also much lower than the paramagnetic part, making the diamagnetic material a poor choice for sensing purposes. A thermal camera is too expensive for OCTs and the only contact temperature measurement that keeps OCT advantages has to be FOS based, as Willsch et al. proposed in [134]. Therefore, many temperature compensation methods have been proposed in the literature, and we will mention ten. Methods numbered 6, 7, 8 and 9 are able to compensate for the temperature along the optical path at which the FR accumulates, enabling compensation even in the presence of temperature gradients in the sensor itself:

1. Introduction of controllable DC magnetic field in part of the optical path and using this field for setting the optical quiescent point [135]. Temperature change will shift the optical quiescent point and that will be detected through the DC part of the signal. Feedback will then set up a new appropriate quiescent point, the one that cancels out the Verdet constant temperature change. This method cannot be used for DC magnetic field measurement, and the DC magnetic field actually represents a source of error in this method, as in all AC/DC methods.
2. Temperature-sensitive rotation of measurement head by a bimetal coil is used to compensate for the increase in the Verdet constant by a decrease in the component of the optical path parallel to the field [136]. The field direction has to be known. The introduction of bimetal coil cancels out the best part of FOS advantages.
3. Introduction of temperature-dependent linear retarder into the optical path. The temperature of the sensor head is obtained through the measurement of the retardance of the birefringent plate [137]. With the temperature dependence of the Verdet constant known, an exact value can be used for measured temperature. Similar solutions place temperature-dependent bulk [138,139] or OF [140] retarder into the optical path and compensates by changing the input polarization of light without calculating the temperature.
4. Using two FMs with different temperature dependence on the Verdet constant gives the possibility of monitoring the temperature-dependent ratio of Verdet constants and to measure temperature on that basis [141,142].
5. Growth of crystals with high FR independent of T. Appropriate dopants during the crystal growth of iron garnets can match the temperature dependencies of the Verdet constant and the material saturation magnetization, thereby providing an almost flat temperature response [143]. The composition of temperature-independent FR iron garnet differs from the composition for maximal Verdet constant. Compounding two kinds of rare-earth ions with opposite temperature coefficients is another proposed method [144]. For every manganese content, x, in Cd_1−x_Mn_x_Te (CMT), a crystal light wavelength can be found at which FR is temperature independent [145].
6. Modified AC/DC normalization is proposed for intrinsic FOSs [146], but there are no obstacles for implementation of this method with birefringent bulk FM as well. It is shown that the DC part of the signal is only sensitive to birefringence of the coiled OF and the AC part is beside birefringence, current-sensitive. After splitting the signal in frequency domain, the DC part, which is temperature-dependent through birefringence, is used to compensate the temperature dependence of the AC part by modified normalization: $Output=\frac{P_{AC}}{1+kP_{DC}}$.
7. Interferometric method that simultaneously measures temperature and FR based on a two-beam interferometric configuration in which the temperature is recovered from the phase change of the interferometric fringes and FR from changes in visibility of the interferometric fringes [147]. This method can be applied with any FM but demands high-quality optical components. Great for laboratory work but not very suitable for practical implementation on the field.
8. Using two wavelengths with the same FM, where the Verdet constant has different temperature dependences [148,149]. From the pair of data, both temperature and magnetic field can be calculated. The reported result is quite impressive. In the temperature range from −20 to 100 °C the change in sensor output has been reduced from 18%, uncompensated, to 0.7%, with compensation [148]. This method does not impose restrictions on the frequency bandwidth or type of FM. There is no fundamental obstacle for utilizing it with intrinsic FOS but with intrinsic interferometric solution more care should be paid to the temperature dependence of quarter-wave plate [44].
9. Using OA temperature dependence to measure the temperature and calibrated temperature dependence of the Verdet constant to obtain a temperature-independent result [24]. FR is measured by two optical channels in a reflective configuration, applying ΔΣ normalization with OA canceled out. In this way, a position for the third, transmissive channel is opened, and can be used for OA measurement as depicted in Figure 4.

This method, similarly to 1 and 6, can be applied for AC current measurements only since OA is obtained by integration of the transmissive channel signal. Since the DC magnetic field is a source of error in this method, the magnetic shield around the measurement head can be used to determine the temperature before field measurement. Another solution proposed by Mitsui et al. [71] also uses OA temperature dependence, but shifts the optical quiescent point and reduces the sensitivity opposing the increase in sensitivity due to the Verdet constant increase with temperature decrease. We tried this method with 1 cm long Bi_12_GeO_20_ crystal, but OA temperature dependence dominated the response and we could not compensate in the significant temperature range. This solution can be improved using the idea of Katsukawa et al. [150], who coupled two differently cut Bi_12_SiO_20_ crystals, one with positive and other with negative rotatory power. FR is independent of direction in the FM and the same at both crystals. OA can be controlled by the crystals lengths and OA can be annulled or reduced to the level suitable for temperature compensation by the optical quiescent point shift.

10. Integral approaches are able to solve overall temperature dependence by combining the various contributions to the temperature dependence [151] or by neural network training [152].

## 6. Choice of the Faraday Material

Choice of the FM is crucial for magnetic field FOSs but less important for OCTs, since magnetic ring concentrators or an increased number of windings increase sensitivity and weak currents are measured by other means. A magnetic field FOS has to be extrinsic, with bulk FM placed in a mechanically stable dielectric housing if we want to keep all the benefits. Bulk solid-state FMs can be divided into glasses and crystals. Crystals have higher FR [16] but their application is constrained to extrinsic FOS. Another division can be made according to the magnetic nature of the FM [132]. Glasses are isotropic, cheaper and easier to produce in different shapes and lengths and can be utilized to produce OFs for intrinsic FOSs. Spun OFs possessing the FE [151,153,154,155] with a reported sensitivity of $100\;\mu A\;\mathrm{rms}/\sqrt{\mathrm{Hz}}$ [156] are commercially available. A decision about the best FM is beyond our reach, and comparative study of FMs for sensing is welcomed. Ideal bulk FM for sensing purposes should exhibit no Pockels effect and no birefringence. OA can be canceled out in absence of the Pockels effect and birefringence. FR should be as high as possible, but wavelength should be chosen for the maximum of magneto-optical quality rather than for Verdet constant maximum. Example of a convenient form of displaying FM properties is presented in Table 1. Knowledge of the dispersion relations for the Verdet constant, magneto-optical quality, χ, (as measured by Kruk and Mrozek [157]) and optical rotatory power, ρ, would be even better.

Much more data about the Verdet constant can be found in the literature (or calculated from presented data) [43,125,159,161,162,163,164,165,166,167,168,169,170,171,172,173,174,175,176,177,178,179,180,181], but the value is usually given for a single wavelength and other figures of merit are often missing. Differences in reported data are understandable because of the high sensitivity of the parameters-to-dopants concentration. In glasses, V increases with an increase in rare-earth dopant concentration [61,182], but absorption also increases. Note that FR can be even twice smaller in OF compared to bulk material [17].

Among bulk crystals, Cd_1−x_Mn_x_Te has the highest Verdet constant. In CMT at room temperature the FE is linear and has no saturation up to large fields, $H=24\times 10^{6}\;A/m$ [60]. Additionally, for manganese concentration x > 0.45, the FE does not depend on the magnetic field frequency up to l GHz. The Verdet constant increases with the increase in manganese share, x [169]. But with the increase in x, absorption also increases, and the lattice of CMT is more strained, making it hard to manufacture crystals without defects. Crystals also become more and more fragile. CMT possesses the Pockels effect [43], and that complicates its usage for magnetometry, but with the concentrator solution the crystal is partially shielded by the ferromagnet. The lowest measurable field reported for 1.3 mm long Cd_0.57_Mn_0.43_Te is 73.2 A/m, which corresponds to 58.3 μT in vacuum [60].

Non-reciprocity of the FE has been used to increase total FR in resonant structures since 1964 [183,184,185,186] (note the couple of resonances for the couple of refraction indexes). Besides sensing, FR is used for Faraday isolators and a lot of work has been done on increasing total FR. Gigantic FR has been reported for thin films [187,188,189,190], magneto-optical photonic crystals [20,191,192,193] and ferrofluids [22,194,195,196,197]. A few exotic structures possessing or mimicing FR have been reported [198,199,200,201,202,203,204,205,206,207,208,209]. A Verdet constant three orders of magnitude higher than one of CMT has been achieved [189]. Yet most of these structures can be made only as thin films and appropriate figures of merit for sensing purposes are total rotation per field, which is Vl product, and transmittance. Specific rotation, important for Faraday isolators and expressed in °/μm, is given at the point of saturation magnetization of the material. Although FR is approximately linear, in ferrimagnetic materials the FA can exhibit hysteresis [187,210] and data for small fields would be better information for sensing applications. Additionally, it is often not clear what the maximum optical length available is. We will compare three promising FMs all at a HeNe laser wavelength in Table 2. Two of them, Cd_0.57_Mn_0.43_Te and (TmBi)_3_(FeGa)_5_O_12_ on Gd_3_Ga_5_O_12_, we used and measured similar data as reported in literature. Martinez et al. reported interesting results for ferrofluid [22], the third FM we will compare. Besides high FR, no existence of linear birefringence in ferrofluids has been reported yet, and ferrofluid does not exhibit Pockels effect, or it is negligible.

Assuming the same measurement conditions as with CMT measurements, the minimal detectable field for (TmBi)_3_(FeGa)_5_O_12_ would be 5 μT and 1.2 μT for ferrofluid.

Spatial resolution in the longitudinal dimension is defined by FM thickness and in the transversal direction by light beam diameter. With thin films with gigantic FR, submillimeter resolution can be achieved in all three dimensions.

## 7. Discussion

FOSs based on the FE can be designed either as a magnetic field sensor or as an electric current sensor. Entanglement of measurement techniques and limitations they impose are a design problem but are solvable for a lot of specific applications. If, for example, a short pulse current ought to be measured, heterodyne detection is excluded because of frequency range limitations but Δ/Σ normalization can be applied with two wavelengths of light used for measurement range expansion. For a short pulse current this is good enough since perturbations from thermal and mechanical domains are too slow. The magnetic concentrator is redundant since the pulse current is the dominant source of the field.

Currently, three configurations are mostly researched:

1. FMs with additional optics and OFs, usable for both magnetometry (blue background in Table 3) and current sensing (green background in Table 3);
2. Magnetic ring concentrator with measurement head for magnetic field measurement placed into the air gap;
3. Reciprocal reflection Sagnac interferometer with closed-loop heterodyne detection.

Properties of these configurations are summarized in Table 3. 

Fully dielectric, mechanically stable measurement head together with Δ⁄Σ normalization ensure that frequency bandwidth depends only on optoelectronic block and FM for extrinsic type. A GHz frequency range have been reported for TGG and CMT [211], and 700 MHz for YIG [212] crystals. Bandwidth depends on FM thickness and dopant concentration. FOSs cannot equal FM bandwidth [213], but device bandwidths of 10 MHz [212] or more [60] have been reported, enabling FE-based sensors to compete for exotic applications [211,214]. The magnetic concentrator spoils bandwidth of extrinsic OCTs, but 10 kHz is easily achievable. Intrinsic OCT beside carrier frequency has an additional limit imposed by the time of flight through the sensing OF [215] in the range of hundreds of MHz.

ΔΣ normalization suppresses light source polarization and intensity fluctuations for extrinsic FOS. Temperature compensation methods numbered 8 and 10 are applicable for every type of FM and method 9 can be applied if the FM possesses OA. The only crosstalk left to be concerned about are from mechanical domain and electrical domain if the FM used possesses the Pockels effect. With thin films with gigantic FR, submillimeter resolution in all three dimensions is possible.

The nonlinear transfer function is such that sensitivity decreases with field increase and the upper limit of the measurement range depends on desired performance. Widening of the measurement range can be done by using two wavelengths. A more expensive interferometric setup combined with heterodyne detection enables a linear response and wide measurement range, but limits frequency bandwidth to frequencies below the modulation frequency of the carrier.

The lowest measurable field is hard to estimate since it also depends on photodiode noise, frequency range and the rest of electronics besides FM. CMT is experimentally proven to operate in the μT range. Further improvements rely on new and better FMs. The possibility of constructing a fully dielectric and passive measurement head with a good spatial resolution and a wide frequency range is the fundamental advantage of FE magnetometry.

An extrinsic OCT is a magnetic field measurement head placed into the air gap of a magnetic ring concentrator. An openable concentrator can be made in the form of a current clamp. All solutions of magnetic field FOSs are applicable here as well. A ferromagnetic concentrator introduces hysteresis and additional nonlinearity but does not affect temperature dependence. Simplicity, easy maintenance, safety and portability are the main advantages of this solution.

An intrinsic solution is the best for static OCTs in power systems with proven reliability [216]. Sensitivity can be controlled by the number of OF coils around the conductor. A wide measurement range, linear response and normalization can be achieved by heterodyne detection. Cost-effective temperature compensation for AC currents can be done by modified AC/DC normalization (method number 6). Vibrations are again the main source of error.

## 8. Conclusions

Replacing sensor energy flow from the electrical domain to the optical domain (photons instead of electrons) bears many advantages when the sensor is measuring physical quantities from the magnetic and electrical domain. It also bears problems related to cross-sensitivity to physical quantities from the thermal and mechanical domains. Measurement methods developed for FOSs based on the FE that diminish these cross-sensitivities are presented together with methods for the normalization of optical signals, widening of the measurement range and obtaining linear responses.

From the point of view of a specific FOS application, methods for achieving the desired performance as discussed in Section 3, Section 4 and Section 5 can be mutually exclusive and interdependent, preventing any particular design from becoming the universal measuring solution. However, for any given practical FOS application effective solutions exist.

Three main directions of research are described. A reciprocal reflection Sagnac interferometer is currently the most prosperous configuration with an important application in electric power grid monitoring.

Price and availability of FMs and optical components will determine the commercial success of FE-based FOSs. Sensing is just one of many FE applications, and regardless of FOS market status, Michael Faraday left us a most interesting legacy.

## Figures and Tables

**Table 1:** Properties of several Faraday materials.

Faraday Material	Glass/Crystal	|V|(rad/Tm)/λ (nm)	χ (rad/T)/λ (nm)	ρ (rad/mm)/λ (nm)	Linear Birefringence	Pockels Effect
Bi_12_GeO_20_ [46,158]	Crystal	72/633	2.1/633	0.6065/633 (T = 293 K)	No	Yes
Cd_0.57_Mn_0.43_Te [60]	Crystal	3140/633	7.85	No	No	Yes
BK-7 glass [159]	Glass	4.3/633	>8.6	No	Yes	Yes
Tb^3+^-dopedGeO_2_-B_2_O_3_-Al_2_O_3_Ga_2_O_3_ [160]	Glass	119/633	>2.4	No data	No	No data

**Table 2:** Comparison of three Faraday structures for sensing applications.

Faraday Material	OPL	|V| (rad/Tm)	$\;\alpha \;(cm^{-1})$	Vl (rad/T)	α*l*
Cd_0.57_Mn_0.43_Te [60]	1.3 mm	3140	4	4	0.52
(TmBi)_3_(FeGa)_5_O_12_ on GGG [189]	60 μm	$1.25\times 10^{6}$	700	75	4.2
Ferrofluid [22]	2.8 mm	122.43×10^3^	2.9	311	0.74

**Table 3:** Comparison of three FOS configurations.

	Magnetometry	Current Sensing	Current Sensing
	Current Sensing		
Configuration	1	2	3
FOS type	Extrinsic	Extrinsic	Intrinsic
Portability	Yes	Yes	No
The best normalization method available	ΔΣ	ΔΣ	By heterodyne detection
Linear response	No	No	Yes
Measurement range	$\mathrm{Limited}\;\mathrm{by}\;B=\frac{\pi }{4Vl}$	$\mathrm{Limited}\;\mathrm{by}\;B=\frac{\pi }{4Vl}$	Wide, limited by phase modulator
Temperature compensation methods available (as listed in Section 4)	4, 5, 6 (for birefringent FM), 8, 9 (for FM that possesses OA) and 10	4, 5, 6 (for birefringent FM), 8, 9 (for FM that possesses OA) and 10	3, 6, 8 and 10
Sensitivity	Determined by magneto-optical quality of FM and detector noise	Determined by magneto-optical quality of FM, detector noise and concentrator properties	Determined by the Verdet constant of OF, number of OF coils and detector noise
Limiting factor for frequency range	FM and optoelectronic block	Concentrator properties	Phase modulator frequency or time of flight (for long-sensing OF)
Full dielectric measurement head	Yes	No	Yes
Main problem to be solved	Low modulation depth	Concentrator hysteresis	Temperature- and vibration-dependent birefringence of sensing OF
	Sensitivity to other magnetic field sources		
Main advantages	Totally dielectric measuring head	Portability and simplicity	Linear response and wide measurement range
	No EMI		
Possible application	High-speed magnetic field measurement with good spatial resolution	Portable OCT for power system monitoring	Static OCT for smart grids
	Pulse current measurement		
Cost	Low	Moderate	High

## Appendix A. Linear State of Polarization Rotation Angle for Isotropic, Dielectric Material

Any medium that rotates the plane of polarization of light has the tensor of dielectric permittivity in the form [27,30]:

(A1) $$\varepsilon =\left(\begin{array}{ccc} \varepsilon_{xx} & j\varepsilon_{xy} & 0\\ -j\varepsilon_{xy} & \varepsilon_{yy} & 0\\ 0 & 0 & \varepsilon_{zz} \end{array}\right),$$

where all terms are real if we neglect absorption. Expanding off-diagonal terms in the ε tensor to the first order in *B* gives [51]:

(A2) $$\varepsilon_{xy}=\varepsilon_{xy}^{\left(0\right)}+\varepsilon_{xy}^{\left(1\right)}B,\;$$

where $\varepsilon_{xy}^{\left(0\right)}\neq 0$ means that media exhibit OA and $\varepsilon_{xy}^{\left(1\right)}\neq 0$ that media exhibit FR. Both terms are antisymmetric,

(A3) $$\varepsilon_{yx}^{\left(0\right)}=-\varepsilon_{xy}^{\left(0\right)},\;\varepsilon_{yx}^{\left(1\right)}=-\varepsilon_{xy}^{\left(1\right)}$$

but for mediums that possess OA

(A4) $$\varepsilon_{xy}\left(-B\right)\neq -\varepsilon_{xy}\left(B\right).\;$$

Solving the Maxwell equations for dielectric,

(A5) $$rot\vec{E}=-\frac{\partial \vec{B}}{\partial t}$$

(A6) $$rot\vec{H}=\frac{\partial \vec{D}}{\partial t}$$

(A7) $$div\vec{D}=0$$

(A8) $$div\vec{B}=0,$$

with the assumed connections between the electric displacement field $\vec{D}$, electric field $\vec{E}$, magnetic flux density $\vec{B}$ and magnetic field $\vec{H}$ in the form:

(A9) $$\vec{D}=\varepsilon \vec{E}$$

(A10) $$\vec{B}=\mu_{0}\vec{H},\;$$

for the lightwave traveling in the *z* direction,

(A11) $$\left(\begin{array}{c} E_{x}\\ E_{y}\\ B_{x}\\ B_{y} \end{array}\right)=\left(\begin{array}{c} E_{0x}\\ E_{0y}\\ \mu_{0}H_{0x}\\ \mu_{0}H_{0y} \end{array}\right)exp\left[j\left(\omega t-kz\right)\right],\;E_{z}=const.,\;$$

with:

(A12) $$\left(\begin{array}{c} D_{x}\\ D_{y}\\ D_{z} \end{array}\right)=\left(\begin{array}{c} \varepsilon_{xx}E_{x}+j\varepsilon_{xy}E_{y}\\ \varepsilon_{yy}E_{y}-j\varepsilon_{xy}E_{x}\\ \varepsilon_{zz}E_{z} \end{array}\right)$$

(A13) $$rot\vec{E}=\left(\begin{array}{c} \frac{\partial E_{z}}{\partial y}-\frac{\partial E_{y}}{\partial z}\\ \frac{\partial E_{x}}{\partial z}-\frac{\partial E_{z}}{\partial x}\\ \frac{\partial E_{y}}{\partial x}-\frac{\partial E_{x}}{\partial y} \end{array}\right)=\left(\begin{array}{c} jkE_{0y}\\ -jkE_{0x}\\ 0 \end{array}\right)exp\left[j\left(\omega t-kz\right)\right]$$

(A14) $$-\frac{\partial \vec{B}}{\partial t}=\left(\begin{array}{c} -\mu_{0}j\omega H_{0x}\\ -\mu_{0}j\omega H_{0y}\\ 0 \end{array}\right)exp\left[j\left(\omega t-kz\right)\right]$$

(A15) $$rot\vec{H}=\left(\begin{array}{c} \frac{\partial H_{z}}{\partial y}-\frac{\partial H_{y}}{\partial z}\\ \frac{\partial H_{x}}{\partial z}-\frac{\partial H_{z}}{\partial x}\\ \frac{\partial H_{y}}{\partial x}-\frac{\partial H_{x}}{\partial y} \end{array}\right)=\left(\begin{array}{c} jkH_{0y}\\ -jkH_{0x}\\ 0 \end{array}\right)exp\left[j\left(\omega t-kz\right)\right]$$

(A16) $$\frac{\partial \vec{D}}{\partial t}=\left(\begin{array}{c} j\omega \varepsilon_{xx}E_{0x}+j\varepsilon_{xy}\left(j\omega \right)E_{0y}\\ j\omega \varepsilon_{yy}E_{0y}-j\varepsilon_{xy}\left(j\omega \right)E_{0x}\\ 0 \end{array}\right)exp\left[j\left(\omega t-kz\right)\right],$$

gives:

(A17) $$\left(\begin{array}{cccc} 0 & k & \mu_{0}\omega & 0\\ k & 0 & 0 & -\mu_{0}\omega \\ -\omega \varepsilon_{x} & -j\varepsilon_{xy}\omega & 0 & k\\ j\varepsilon_{xy}\omega & -\omega \varepsilon_{yy} & k & 0 \end{array}\right)\left(\begin{array}{c} E_{0x}\\ E_{0y}\\ H_{0x}\\ H_{0y} \end{array}\right)=\left(\begin{array}{c} 0\\ 0\\ 0\\ 0 \end{array}\right)$$

and by eliminating the magnetic field we obtain:

(A18) $$\left(\begin{array}{cc} \varepsilon_{xx}-\frac{k^{2}}{\mu_{0}\omega^{2}} & j\varepsilon_{xy}\\ -j\varepsilon_{xy} & \varepsilon_{yy}-\frac{k^{2}}{\mu_{0}\omega^{2}} \end{array}\right)\left(\begin{array}{c} E_{0x}\\ E_{0y} \end{array}\right)=\left(\begin{array}{c} 0\\ 0 \end{array}\right).$$

Condition for nontrivial solutions:

(A19) $$\det\left(\begin{array}{cc} \varepsilon_{xx}-\frac{k^{2}}{\mu_{0}\omega^{2}} & j\varepsilon_{xy}\\ -j\varepsilon_{xy} & \varepsilon_{yy}-\frac{k^{2}}{\mu_{0}\omega^{2}} \end{array}\right)=0,\;$$

reduces to:

(A20) $${\left(k^{2}\right)}^{2}-\mu_{0}\omega^{2}\left(\varepsilon_{xx}+\varepsilon_{yy}\right)k^{2}+{\left(\mu_{0}\omega^{2}\right)}^{2}\left(\varepsilon_{xx}\varepsilon_{yy}-\varepsilon_{xy}{}^{2}\right)=0,$$

and gives two possibilities for wavenumber:

(A21) $${\left(k_{\pm }\right)}^{2}=\frac{1}{2}\mu_{0}\omega^{2}\left[\left(\varepsilon_{xx}+\varepsilon_{yy}\right)\pm \sqrt{{\left(\varepsilon_{xx}-\varepsilon_{yy}\right)}^{2}+4\varepsilon_{xy}{}^{2}}\right].\;$$

For isotropic material,

(A22) $$\varepsilon_{xx}=\varepsilon_{yy}=\varepsilon_{zz}=\varepsilon_{d},\;$$

two orthogonal circular modes exist:

(A23) $$\left(\begin{array}{c} E_{x}^{1}\\ E_{y}^{1} \end{array}\right)=E_{0}\left(\begin{array}{c} 1\\ -j \end{array}\right)exp\left[j\left(\omega t-k_{+}z\right)\right]\;\mathrm{Right}\;\mathrm{circularly}\;\mathrm{polarized}\;\mathrm{mode},$$

(A24) $$\left(\begin{array}{c} E_{x}^{2}\\ E_{y}^{2} \end{array}\right)=E_{0}\left(\begin{array}{c} 1\\ -j \end{array}\right)exp\left[j\left(\omega t-k_{-}z\right)\right]\;\mathrm{Left}\;\mathrm{circularly}\;\mathrm{polarized}\;\mathrm{mode},$$

with circular retardation

(A25) $$\Delta k=\sqrt{\mu_{0}\omega^{2}\varepsilon_{d}}\left(\sqrt{1+\frac{\varepsilon_{xy}}{\varepsilon_{d}}}-\sqrt{1-\frac{\varepsilon_{xy}}{\varepsilon_{d}}}\right)\approx \sqrt{\frac{\mu_{0}\omega^{2}}{\varepsilon_{d}}}\varepsilon_{xy}=\sqrt{\frac{\mu_{0}\omega^{2}}{\varepsilon_{d}}}\left(\varepsilon_{xy}^{\left(0\right)}+\varepsilon_{xy}^{\left(1\right)}B\right).\;$$

and the rotation of the plane of polarization of light is:

(A26) $$\theta =\frac{\left(k_{+}-k_{-}\right)z}{2}=\frac{\left(k_{R}-k_{L}\right)z}{2}=\frac{\Delta kz}{2}=\frac{1}{2}\sqrt{\frac{\mu_{0}\omega^{2}}{\varepsilon_{d}}}\left(\varepsilon_{xy}^{\left(0\right)}+\varepsilon_{xy}^{\left(1\right)}B\right)z=\theta_{0}+VBz,\;$$

where $\theta_{0}$ is optical activity.

## Appendix B. Transfer Function for Reciprocal Reflection Interferometer with Heterodyne Detection

Interferometer output is:

(A27) $$U=\frac{\beta P_{0}}{2}\left(1+\cos\left(2\theta +\varphi_{0}\cos\left(\omega_{m}t\right)\right)\right)$$

where β is a constant that includes all optical losses, as well as the optoelectronic conversion efficiency, $P_{0}$ is the light source power, θ is FA, $\varphi_{0}$ is the amplitude of phase modulation and $\omega_{m}$ is the phase modulator circular frequency.

(A28) $$U=\frac{\beta P_{0}}{2}\left(1+\cos2\theta \cos\left(\varphi_{0}\cos\left(\omega_{m}t\right)\right)-\sin2\theta \sin\left(\varphi_{0}\cos\left(\omega_{m}t\right)\right)\right)$$

The output can be expanded as:

(A29) $$U=\frac{\beta P_{0}}{2}\left(1+\cos2\theta \left(J_{0}\left(\varphi_{0}\right)+2\sum_{k=1}^{\infty }{\left(-1\right)}^{k}J_{2k}\left(\varphi_{0}\right)\cos\left(2k\omega_{m}t\right)\right)-2\sin2\theta \sum_{k=1}^{\infty }J_{2k-1}\left(\varphi_{0}\right)\sin\left(\left(2k-1\right)\omega_{m}t\right)\right)$$

where $J_{k}\left(\varphi_{0}\right)$ are Bessel functions of the first kind and *k*-th order. By filtering around the $\omega_{m}$ signal

(A30) $$U_{1}=-\beta P_{0}\sin2\theta J_{1}\left(\varphi_{0}\right)\sin\left(\omega_{m}t\right)$$

is obtained and by filtering around the $2\omega_{m}$ signal

(A31) $$U_{2}=\beta P_{0}\cos2\theta J_{2}\left(\varphi_{0}\right)\cos\left(2\omega_{m}t\right)$$

is obtained.

Ratio of signal amplitudes is

(A32) $$\frac{U_{1max}}{U_{2max}}=\tan2\theta \frac{J_{1}\left(\varphi_{0}\right)}{J_{2}\left(\varphi_{0}\right)}$$

and calculated FA:

(A33) $$\theta =\frac{1}{2}\mathrm{arctg}\left(\frac{J_{2}\left(\varphi_{0}\right)U_{1max}}{J_{1}\left(\varphi_{0}\right)U_{2max}}\right)$$

is independent of light source power and losses.
